# Novel glass slide preparation system for single DNA molecules analysis

**DOI:** 10.1080/13102818.2014.901687

**Published:** 2014-05-02

**Authors:** Alexander Zarkov, Stoyno Stoynov, Marina Nedelcheva-Veleva

**Affiliations:** ^a^ Institute of Molecular Biology “Roumen Tsanev”, Bulgarian Academy of Sciences, Sofia, Bulgaria

**Keywords:** fluorescent microscopy, single DNA molecules, DNA stretching, glass slide surface modification

## Abstract

Here we propose an easy to build up and apply glass slide preparation system for single DNA molecules stretching. It is based on fast and simple coating of a solid glass with a cocktail of acrylic monomers that are easily polymerized via ultraviolet illumination. The acrylated slides are used to successfully stretch DNA molecules in a broader pH range compared to that of the commonly used amino-silanes. Moreover, the single DNA molecules that are stretched on the acrylated slides give a brighter and more photostable signal when visualized under a fluorescent microscope.

## Introduction

The ability to optically visualize individually stretched DNA molecules dyed by different fluorophores has advantaged the genomic analyses in recent years. This approach complements the classical biochemical techniques to obtain new data related to DNA mechanical properties, polymeric behaviour, DNA–protein interactions, etc. That is why, stretching of DNA fragments onto a solid surface is one of the hottest areas of technological development to simplify and improve the procedure.

Recently, based on single DNA techniques, many novel types of analysis have been developed. Via dynamic molecular combing, stretching and aligning of entire genomes, the precise measurement of hybridized DNA probes has been improved.[1,2] By this technique, the transcription activity of RNA polymerase on combed DNA by direct visualization of newly synthesized fluorescent RNAs has been studied.[3] Molecular combing has also been used to develop an approach to study DNA replication. It allows for a genome-wide analysis of the spatial and temporal organization of replication units and replication origins in a sample of genomic DNA [4] and studies of replication fork progress and direction.[5]

Many different stretching procedures have been developed to facilitate a particular type of analysis. Usually, DNA molecules are attached with one of their ends to a pre-treated glass surface. They are extended by means of application of different weak forces. Some techniques are based on stretching of DNA molecules in an aqueous solution by their movement at the interface between air and a treated glass.[6–8] Others (known as “molecular combing techniques”) use the force of spin-stretching,[9] air-blowing a droplet,[10] filter paper absorption,[11] and dipping a substrate into a solution and then lifting it up (dynamic molecular combing) [2,12] for stretching and aligning of individual DNA molecules.

Different types of fluorophores have been developed and applied in single molecule approaches. There are varieties of microparticles (phosphors, quantum dots, nanocrystals, etc.) and dyes that bind nucleic acids directly. The latter are mainly fluorophores similar to fluorescein (rhodamine, carboxyfluorescein, cyanine dyes). Currently, the most widely used ones are the cyanine dyes, as they increase their fluorescence emission up to 1000 times when intercalated into nucleic acids to produce high fluorescence quantum yields.[13] They are basically divided into two families of monomeric cyanine dyes, e.g. TO-PRO™, YO-PRO™, PO-PRO™; and dimeric cyanine dyes, e.g. TOTO®, YOYO®, BOBO®, POPO™ (Invitrogen™).[14–16]

In order to ensure stable attachment of DNA molecules, the surface of the solid glass is usually modified. There are several ways to achieve that: ether by chemical modification of the glass or by coating it with a polymer.[6] One of the basic requirements is the surface to be extremely clean and free of background fluorescence. The other condition is the polymer that is to be applied not to affect the assay. Such a specific binding is observed at pH 5.5 (MES (2-[N-morpholino]ethanesulphonic acid) buffer) on surfaces coated with silane and exposing various groups: vinyl-silane (–CH=CH_2_),[7] amino-silane (e.g. γ-amino-triethoxysilane (NH_2_–(CH_2_)_3_–Si(OCH_2_CH_3_)_3_),[6] etc. Some other surfaces were also proven to be appropriate for single molecule techniques, such as polylysine, polyhistidine, polystyrene, etc. by means of fitting the choice of pH for binding and stretching.[6]

Our study aims to create an easy to prepare and apply protocol for glass slide preparation to be used for single DNA molecule techniques. We developed a protocol that uses a cocktail of acrylates and demonstrated that our glass slides can be used for successful stretching of DNA molecules in a broader pH range compared to that of the routinely used amino-salanes. In addition, the single DNA molecules that are stretched on the acrylated slides emit a brighter and more photostable signal when visualized under a fluorescent microscope.

## Materials and methods

### Reagents and slides

The dimeric cyanine nucleic acid dye YOYO-1 iodide (Invitrogen, N-7565) was used for staining of single DNA molecules.

The stretching of fluorescent DNA fragments was done onto a pre-treated solid surface. Three types of glass slides were used: the commercially available ready-to-use microscope glass slides silanized with aminoalkylsilane (SIGMA, S4651) and glass slides covered with APTES (aminopropyl-trimethoxysilane; SIGMA, 281778) and acrylates (ethyl methylethylmetacrylate, metacrylate and dimethacrylate – A Polymer, EzFlow Nail Systems).

### Glass slide preparation

The glass slides are cleaned by soaking for 3–4 h at room temperature in a solution of HNO_3_ and HCl in a 2:1 ratio. The procedure is carried out in a hood. After three washing procedures in deionized ultrapure water, the glass slides are drained on air in a vertical position.

The silanization procedure by means of aminopropyl-trimetoxysilane (APTES) is based on a previously described protocol.[17] The slides are exposed to air that is rich in APTES evaporations. The slides are positioned upright in a glass container. Next to them, an open small vessel with 5 μL of APTES is placed. The container is then placed in a glass chamber that is incubated at 100 °C for 30 min.

The preparation of glass slides covered with acrylates (ethyl methylethylmetacrylate, metacrylate and dimethacrylate) is carried out as follows. A synthetic brush is dipped into dimethyltolyamine (“High definition”, EzFlow Nail Systems) and then into a small vessel that contains the powder of acrylates. After mixing, the substance is spread onto the clean glass slide in a fine layer. The excess acrylates are removed from the slide by means of a microfibre sterile cloth. The glass slide is then ultraviolet (UV) illuminated (366 nm) for 5 min in order to allow the acrylates to polymerize.

### DNA staining

To prepare single DNA fragments, total genome DNA from *Saccharomyces cerevisiae* was lightly digested via Bgl I restriction endonuclease (New England BioLabs). The isolation of DNA from the yeast *S. cerevisiae* was carried out via a standard procedure.[18] For DNA dying reactions, a ratio of 1:20 of YOYO-1 molecules to DNA base pairs was applied. The reactions were carried out in 150 mmol·L^−1^ MES (2-morpholinoethanesulphonic acid) buffer (pH 5.5) or TE (10 mmol·L^−1^ Tris, pH 8.0 and 1 mmol·L^−1^ EDTA) buffer (pH 7.5) for 30 min at room temperature in a dark place. Twenty per cent β-mercaptoethanol was added before microscopy in order to reduce the photobleaching by scavenging oxygen from the solution.[19]

### Microscopy

The stretching of DNA molecules was carried out following a procedure that is based on a published protocol.[8] Four microlitres of the stained DNA solution are placed onto the pre-treated or ready-to-use microscope glass slide. A coverslip is carefully positioned to barely contact the drop but not the glass slide. Then it is slightly pushed towards the glass slide in order to carefully spread the drop. Then the two glasses are slightly pressed to one another for 10–15 s. Small wafer bands are positioned on the edges of the coverslips. Microscopy was carried out on an Axiovert 200M, Zeiss inverted microscope by EC-Plan Neofluar 100×/1,3 oil-immersion objective. Pictures were taken by an AxioCam MRm charge-coupled device (CCD) camera, Carl Zeiss. The samples were constantly illuminated for 171 s under a green fluorescent filter: Filter set 38 HE, Zeiss (excitation BP 470/40; beamsplitter FT 495; emission BP 525/50). Images were acquired every 14 s at 450 ms exposure time and were processed by Carl Zeiss AxioVision Rel.4.7 software. ImageJ software was used for measurements of the fluorescent signal intensities for 10 stretched DNA molecules. The corresponding background fluorescence was subtracted. Presented results are mean values. The standard error of the mean is indicated as error bars.

## Results and discussion

### Preparation of glass slides for single molecule techniques

Our aim was to develop an easy to use and apply method for preparation of microscopic glass slides to use for visualization of single DNA molecules stretched on a solid surface. For that purpose we used a cocktail of acrylates (ethyl methylethylmetacrylate, metacrylate and dimethacrylate – A Polymer, EzFlow Nail Systems) in order to modify the glass slide surface by coating it with a polymer and to ensure stable DNA fragments attachment and stretching. First, to guarantee that the glass slides themselves lack background fluorescence that may compromise the molecule analysis, we cleaned the glass slides carefully with a solution of HNO_3_ and HCl in a 2:1 ratio (see “Materials and methods” section). Then the glass slides were covered with a powder of acrylates diluted in dimethyltolyamine. A thin layer of the solution was placed on one of the sides of the glass slide and the excess solution was removed by rubbing with a microfiber sterile cloth that could not contaminate the layer with fibres. In order to achieve a solid polymer cover, the treated glass was UV illuminated (366 nm) for 5 min. After this short procedure the acrylated glass slides are ready to use or can be stored for 4–5 months in a dust-proof container. The procedure is described in detail in “Materials and methods” section.

In order to compare the qualities of our acrylated glass slides with other types of standard coatings, we also prepared glass slides silanized with APTES by following the procedure of Yokota et al.[17] For our study we also used commercially available ready-to-use microscope glass slides silanized with aminoalkylsilane (for details see “Materials and methods” section).

### Stretching and imaging of single DNA molecules pre-treated with fluorescent dyes

To test our acrylated glass slides we used fragmented total genome DNA from *S. cerevisiae.* Before stretching, we carried out dying reactions by incubation of DNA with the dimeric cyanine nucleic acid dye YOYO-1 iodide. It possesses high molar excitation coefficient (117,000 L·mol^−1^·cm^−1^) and quantum yield for DNA complexes *QY* = 0.52, which gives the dye high sensitivity and affinity for nucleic acids. The dying reactions are carried out in 150 mmol·L^−1^ MES buffer (pH 5.5) or TE buffer (pH 7.5) [16] (for details see “Materials and methods” section). The stretching of DNA molecules was carried out following a procedure that is based on a published protocol.[8] To visualize fluorescent single DNA molecules we used an Axiovert 200M, Zeiss inverted microscope and EC-Plan Neofluar 100×/1,3 oil-immersion objective. As the spectral characteristics of YOYO-1 iodide are maximum wavelengths of absorption, excitation and emission spectra respectively 491, 488 and 509 nm, the microscopy was carried out under a green fluorescent filter set.

**Figure 1.**
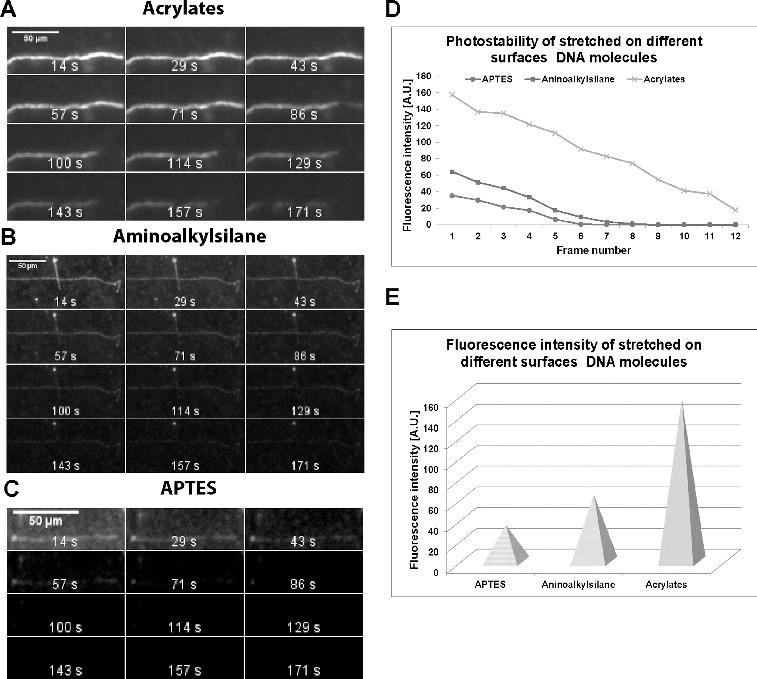
Effect of slide surface treatment on fluorescence intensity and photostability. Pictures present time-lapse experiments of single DNA molecules stained with YOYO-1 in 150 mmol·L^−1^ MES buffer (pH 5.5). Acrylated glass slides (A). Aminoalkylsilanized glass slides (B). APTES-covered glass slides (C). Intensity of the emitted fluorescence of stained DNA molecules (D) for each time-frame (subtracting the background fluorescence). Abscissa: individual time-frames of the 3 min interval are presented. Ordinate: fluorescence intensity in arbitrary units. The fluorescence intensity of molecules stretched on APTES-, aminoalkylsilane- and acrylates-treated glass slides (corresponding to the first time-frame) is given as individual pyramids (E).

The intensity of the emitted fluorescent signal of the stretched molecules and the photostability of that signal were analysed. First, the YOYO-1 staining was carried out in 150 mmol·L^−1^ MES buffer (pH 5.5) only, but molecules were stretched on the acrylated, ready-to-use aminoalkylsilanized and APTES-covered glass slides. The samples were constantly illuminated for about 3 min under the green microscopic filter set. Pictures were acquired every 14 s by applying 450 ms exposure time. The data obtained for DNA molecules stretched on the three types of polymer-covered glass slides, were documented and analysed (Figure 1(A)–(C)). By means of ImageJ software, the intensity of fluorescent DNA–dye complex signal was measured for each time-frame (subtracting the background fluorescence). The summarized data are presented as diagrams in Figure 1(D). The fluorescence maximum (corresponding to the first time-frame) is shown in Figure 1(E). The results undoubtedly indicate that when stretched on acrylated glass slides, the analysed single molecules emit a fluorescent signal more than twice as bright as those on commercial aminoalkylsilane glass slides and more than four times as bright as the molecules on APTES (Figure 1(E)). As a result of this strong fluorescence emission, the same relationship is observed when photostability is studied (Figure 1(A)–(D)). The molecules stretched on our acrylated glass slides emitted fluorescence until the last time-frame. Those on aminoalkylsilane were well visualized until the seventh time-frame (100 s) only. The molecules on APTES were even more photo-unstable. They were visible for just four frames (57 s).

These results indicate that when stretched on acrylated glass slides, the fluorescent single DNA molecules emit not only a strong signal, but also a highly photostable one and can successfully be applied in solid-surface single molecule techniques.

**Figure 2.**
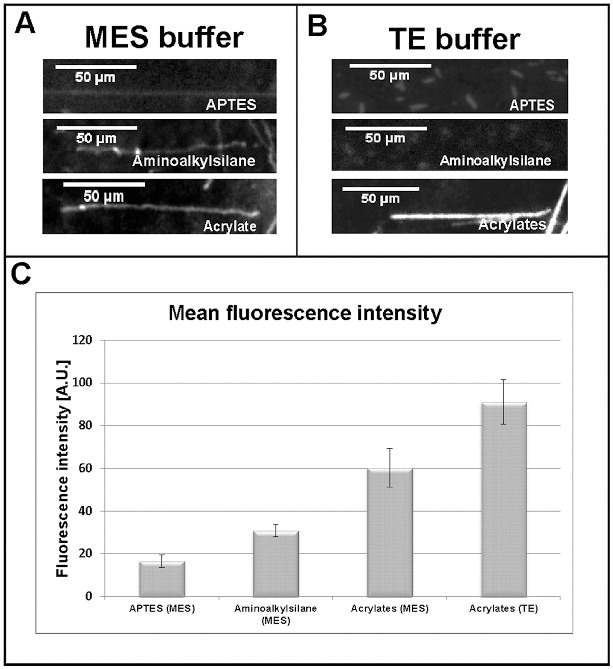
Comparative analysis of single DNA molecules stretched on acrylates-, aminoalkylsilane- and APTES-covered glass slides at different buffer conditions. Pre-stained single DNA molecules at: MES buffer, pH 5.5 (A); TE buffer, pH 7.5 (B). Diagram (C) representing the intensity of the emitted fluorescent signal of stretched DNA molecules (*n* = 10).

As TE buffer (pH 7.5) is the most commonly used environment for DNA-related reactions, next we tested whether our acrylated glass slides can be used to stretch DNA molecules dyed in TE buffer. Again, a comparison with stretched molecules on the other two types of silanized glass slides was made. In contrast to molecules dyed and stretched in MES buffer (pH 5.5) (Figure 2(A)), DNA fragments from TE buffer (pH 7.5) reactions, applied on the commercial aminoalkylsilane- and APTES-treated glass slides were not able to stretch at all (Figure 2(B), upper panels). Unlike them, DNA molecules dyed in TE buffer and applied on our acrylated glass slides were perfectly stretched (Figure 2(B), lower panel).

To summarize and quantify our results, we performed an analysis of the intensity of the emitted fluorescence of the successfully stretched DNA molecules, using ImageJ software. The intensity of the fluorescent signal was measured for 10 stretched DNA molecules and the corresponding background fluorescence was subtracted. The data were summarized for every single reaction and average values were calculated. The results are presented as a diagram in Figure 2(C). Interestingly, when dyed in TE buffer, the DNA molecules stretched on acrylated surface emitted a fluorescent signal about 35% brighter compared to those in MES buffer. The difference was even bigger when molecules dyed in TE buffer and stretched on acrylates were compared to those dyed in MES buffer and stretched on aminoalkylsilane or APTES (Figure 2(C)).

The obtained results prove that our acrylated glass slides can be successfully used in a broader area of scientific research when a single DNA molecule technique is applied, as they permit a wider pH range of work. The successful stretching and the high value of emitted fluorescence in TE buffer can find various applications in molecular biology, biophysics and medical diagnostics as most of the biochemical reactions require pH of about 7.

## Conclusions

The results above indicate that our acrylated glass slides are easy to prepare and use. They mediate a bright and photostable signal of stretched single fluorescent DNA molecules dyed with YOYO-1 iodide. The acrylated glass slides are applicable in a wide pH range (tested in MES buffer, pH 5.5 and TE buffer, pH 7.5). That is why, they can successfully be applied and benefit solid-surface single molecules techniques.

## References

[cit0001] Lebofsky R, Bensimon A (2003). Brief Funct Genomics Proteomics.

[cit0002] Michalet X, Ekong R, Fougerousse F, Rousseaux S (1997). Science.

[cit0003] Gueroui Z, Place C, Freyssingeas E, Berge BP (2002). Proc Natl Acad Sci USA.

[cit0004] Herrick J, Bensimon A (1999). Biochimie.

[cit0005] Gongora C, Vezzio-Vie N, Tuduri S, Denis V (2011). Mol Cancer.

[cit0006] Allemand JF, Bensimon D, Jullien L, Bensimon A, Croquette V (1997). Biophys J.

[cit0007] Bensimon A, Simon A, Chiffaudel A, Croquette V (1994). Science.

[cit0008] Henegariu O, Grober L, Haskins W, Bowers PN (2001). Biotechniques.

[cit0009] Yokota H, Sunwoo J, Sarikaya M, van den Engh G, Aebersold R (1999). Anal Chem.

[cit0010] Deng ZX, Mao CD (2003). Nano Lett.

[cit0011] Zhang J, Ma Y, Stachura S, He H (2005). Langmuir.

[cit0012] Geron-Landre B, Roulon T, Desbiolles P, Escude C (2003). Nucleic Acids Res.

[cit0013] Kricka LJ (2002). Ann Clin Biochem.

[cit0014] Hirons GT, Fawcett JJ, Crissman HA (1994). Cytometry.

[cit0015] Rye HS, Yue S, Wemmer DE, Quesada MA (1992). Nucleic Acids Res.

[cit0016] Zarkov A, Vasilev A, Deligeorgiev T, Stoynov S, Nedelcheva-Veleva M (2013). Mol Imaging.

[cit0017] Yokota H, Johnson F, Lu H, Robinson RM (1997). Nucleic Acids Res.

[cit0018] Hoffman CS, Winston F (1987). Gene.

[cit0019] Chan TF, Ha C, Phong A, Cai D (2006). Nucleic Acids Res.

